# Assessment of basic pharmacokinetic parameters of dapagliflozin in TTS formulations in male minipigs

**DOI:** 10.1038/s41598-024-74675-z

**Published:** 2024-10-08

**Authors:** Biernat Paweł, Radosław Balwierz, Dyliński Mieszko, Kołacki Mikołaj, Ewa Micewicz, Bursy Dawid, Łukasz Pogorzelec

**Affiliations:** 1 Biotts SA, Wroclawska 44c St., 55-040 Bielany Wroclawskie, Poland; 2 Institute of Chemistry, University of Opole, Oleska St. 48, 45-052 Opole, Poland

**Keywords:** Transdermal patch, Pharmacokinetics, Type 2 diabetes, Drug formulation, Dapagliflozin, Endocrine system and metabolic diseases, Drug development, Pharmacokinetics

## Abstract

Given the extended time over which diabetes treatment is administered, the transdermal delivery system is anticipated to be a more suitable option for older individuals who may experience difficulty swallowing. The continuous delivery of dapagliflozin and more stable plasma levels are anticipated to reduce the incidence of side effects and the frequency of dosing. The objectives of the study were to determine the safety and plasma pharmacokinetics of dapagliflozin in male minipigs following application of the ointment and skin patch. In the initial phase of the study, the potential for transdermal permeation of dapagliflozin from ointment and transdermal patch to blood plasma of 15 male Göttingen minipigs was investigated. In the subsequent phase, the efficacy of utilising patches of varying strengths and sizes was assessed. The LC/MS method was employed to quantify the concentration of the active substance. The transportation of the studied API to the general circulation and accumulation in tissues were confirmed. The maximum drug concentration (122.99 ng/mL) in plasma was observed on the fourth day of application. The highest calculated C_max_ was 131.91 ng/mL with a mean AUC_0-last_ of 6620.7 ng h/mL. Following transdermal administration, dapagliflozin is excreted in the urine. The trend between urinary dapagliflozin 3-O-glucuronide levels and urinary glucose excretion was also observed. The transdermal patch has been demonstrated to be an effective drug delivery system for dapagliflozin.

**Supplementary Information:**

The online version contains supplementary material available at 10.1038/s41598-024-74675-z.

## Introduction

Transdermal drug delivery is one of the most commonly accepted routes of drug administration by patients. Transdermal therapeutic systems (TTS) offer consistent drug levels, reduced side effects, and pain-free therapy. They allow the use of a wide range of drugs, including hormones, antihypertensive, and analgesics. The use of a sustained-release transdermal system in the treatment of type II diabetes may increase patient comfort and reduce side effects, including those associated with polypharmacy^1,2^. Type 2 diabetes is characterized by *β*-cell loss and insulin resistance, as well as the presence of markers of oxidative stress such as malondialdehyde. Risk factors include age, obesity, and exposure to lipophilic substances such as glucocorticosteroids. Treatment of type II diabetes includes medical therapy, insulin, dietary management, and patient education. Depending on the patient’s needs, antidiabetic drugs can reduce insulin resistance or increase insulin secretion. In recent years, there have been advances in the pharmacotherapy of type 2 diabetes, and new drugs such as liraglutide, semaglutide, and dapagliflozin are now on the market^3–6^. The latter is an oral, highly potent, selective, and reversible inhibitor of sodium-dependent glucose co-transporter 2 (SGLT2) for treating type II diabetes. It is the first SGLT2 inhibitor approved for the treatment of type 2 diabetes. It blocks the reabsorption of glucose in the kidneys, resulting in urinary excretion and lower blood glucose levels. It is well tolerated and can be used as monotherapy or in combination with other medicines on the market. It has a stable safety profile and ongoing studies are monitoring its safety and efficacy. The recommended starting dose is one 5 mg tablet once daily. After oral administration of dapagliflozin, maximum plasma concentration (C_max_) is usually achieved within 2 h in the fasting state. C_max_ and AUC values increase dose-proportionally with increasing dapagliflozin dose over the therapeutic dose range^7–10^. The geometric mean C_max_ and AUC_τ_ of dapagliflozin at steady-state following a 10 mg once-daily dose were 158 ng/ml and 628 ng h/ml, respectively^10^. The absolute oral bioavailability of dapagliflozin after administration of a 10 mg dose is 78%. The mean steady-state volume of distribution is 118 L, indicating extensive extravascular distribution. Dapagliflozin is extensively metabolised, mainly to dapagliflozin 3-O-glucuronide, a more polar inactive metabolite. 61% of orally administered dapagliflozin is excreted as this metabolite in the urine. The mean plasma terminal half-life (t_½_) for dapagliflozin is approximately 12.9 h following a single oral dose of 10 mg. It is worth noting that the exposure of dapagliflozin after oral administration increases in proportion to the increase in dose over the range 0.1 to 500 mg, and its pharmacokinetics remain unchanged after repeated daily dosing for up to 24 weeks^7–11^.

As diabetes treatment is spread over months to years, the transdermal delivery system is expected to be more suitable, particularly in terms of patient compliance. The most common form of non-compliance observed in older people on long-term chronic therapy is underdosing. This is often unintentional, due to forgetfulness or misinterpretation/confusion about the dosing regimen. In addition, older people may have difficulty swallowing, especially if they are experiencing nausea or vomiting. By providing consistent, continuous dapagliflozin delivery and more stable plasma levels, bypassing hepatic first-pass metabolism, transdermal patches are expected to reduce the incidence of adverse events (e.g. genital mycotic infections, hypotension and renal impairment) and reduce dosing frequency, making it easier to achieve optimal therapeutic doses and potentially improving treatment efficacy and compliance^7–10^.

Therefore, transdermal drug delivery systems are a promising approach for treating diabetes, offering improved clinical outcomes compared to conventional dosage forms. Improved drug bioavailability can be achieved, resulting in a lower dosing frequency for diabetic patients than traditional oral medications. Another important advantage is that patients will have a much simpler treatment option where a patch with a specific drug release rate can be applied to the skin. The sustained release profile will also provide improved glycaemic control and predictability of dosing. Bypassing the first-pass effect may also reduce the likelihood of drug-drug interactions through a competitive hepatic mechanism. By providing consistent, continuous transdermal delivery of dapagliflozin and more stable plasma concentrations (bypassing first-pass metabolism in the liver), transdermal patches are expected to reduce the incidence of adverse events and dosing frequency, making it easier to achieve optimal therapeutic doses and potentially improve treatment efficacy and compliance^12–14^.

In addition, the transdermal approach is designed to bypass the UGT1A9 enzyme metabolic reaction observed after oral administration and present in the absorptive region of the intestine, specifically the jejunum, ileum and colon. As dapagliflozin is a P-glycoprotein (P-gp) substrate, the transdermal approach is also expected to bypass P-gp-mediated efflux. Therefore, comparatively less drug per patch would be required to achieve the same efficacy as a 5 mg or 10 mg tablet^7,10^.

It is worth mentioning that according to the Guideline for the Pharmacokinetic and Clinical Evaluation of Modified-Release Dosage Forms (EMA/CHMP/EWP/280/96)^15^, less frequent dosing and a non-oral route of administration justify the development of modified-release dosage forms.

In view of the above, the aim of the study is to determine the safety and pharmacokinetics of dapagliflozin in the blood plasma of male minipigs after application of the ointment and patch to the skin, and to determine the concentration in the skin layers and soft tissues (Step 1), and to assess the efficacy of using dapagliflozin in patches of different strengths (dose of the active ingredient) and sizes (Step 2). It should be noted that there are no studies in the scientific literature to support the possibility of using dapagliflozin in the form of transdermal patches on animal skin.

## Materials and methods

### Tested patches and ointment

Dapagliflozin (Polpharma, Poland) was incorporated into the transdermal vehicle MTC^®^ (Patent No: PL428534A1)^16^ in the form of a non-aqueous emulsion in which the dispersing phase was a mixture of 1,2-propanediol, DMSO and isopropyl alcohol and the dispersed phase was a mixture of lipophilic substrates and chaulmoogra oil. The carrier itself was an ointment base and the prepared ointment had a concentration of 47.2 mg/mL dapagliflozin, then the ointment was incorporated into the reservoir patch in which the concentration of dapagliflozin was 236 mg dapagliflozin per patch or 118 mg per patch, respectively. The reservoir patch was chosen because of the solubility of dapagliflozin in the lipophilic matrix that forms the reservoir of the semipermeable membrane transdermal therapeutic system. Dapagliflozin patches and ointment (the composition of which was compatible with the filling of the patch) have been manufactured by Biotts INC (Poland), which is licensed to manufacture medicines in accordance with GMP standards (The Chief Pharmaceutical Inspectorate licence). The dapagliflozin patches have a patented formula, application number: WIPO ST 10/C PL446900 and WIPO ST 10/C PL446901. Details of the concentrations used in the ointment and the size of the patches and the dose of active ingredient in the patches are given in Table 1 for Step 1 of the study and Table 2 for Step 2 of the study.

### Animals and sample collection

The study was conducted in accordance with OECD principles on Good Laboratory Practice.

The animal model was selected according to the FDA guidelines^17^ and the data presented by Todo^18^. The first step of the study was performed with 15 male Göttingen minipigs (obtained from Ellegard Göttingen Mini pigs A/S, Dalmose, Denmark), and the second step with 12 male Göttingen minipigs (obtained from Ellegard Göttingen Mini pigs A/S, Dalmose, Denmark). The minipigs weighed 16 ± 3 kg: The minipigs were healthy and vaccinated against swine fever, foot-and-mouth disease, and hemorrhagic septicaemia. Minipigs were identified by microchip as temporary identification and by neck collar chain as permanent identification numbers. The minipigs were acclimatized for at least 1 month before the start of the treatment under the conditions recommended by the supplier, i.e. at a temperature of 18–28 °C and a humidity range of 30–70% with a of 12 h light and 12 h dark. The animals were housed in individual stainless steel pens. SDS Standard diets were used and water was provided ad libitum. An amount of approximately 350 g of food (SDS diet) was offered to each minipig approximately 2–3 h after ointment or patch application and maintained for a minimum of 3–4 h per day, after which food was withdrawn. If the application of the ointment or patch induced stress and thus a reduction in food consumption, the food was not withdrawn after 3–4 h but was left in the pen for longer periods. No randomization was used in this study.

As shown in Table 1, the animals were first divided into 6 groups.

#### Group 1

A single intravenous injection (*i.v.*) of dapagliflozin was administered, followed by blood sampling and observation for 2 days. The 3 animals in group 1 were reused in group number 4 after the washout period of 4 days from the last sampling time point (48 h).

#### Group 2

7 days topical application (OD x 7 days) of placebo ointment (D-F-P) followed by occlusion, 3 h later the remaining ointment was removed and the site observed for rash/edema. The 7-day treatment period was followed by a 7-day observation period (total study duration 14 days).

#### Group 3

7 days topical application (OD x 7 days) of dapagliflozin ointment (D-F100) followed by occlusion, 3 h later the remaining ointment was removed and the site observed for rash/edema. The 7-day treatment period was followed by a 7-day observation period (total study duration 14 days).

#### Group 4

A dermal placebo patch (D-TS-P) was applied and left in place for 7 days. The patch was removed on day 8 and the application site was examined for gross pathological changes. Observation continued until day 14.

#### Group 5

Test substance patch (D-TS100) was applied and left on the skin for 7 days. The patch was removed on day 8 and the application site was examined for any gross pathological changes/lesions. Observation continued until day 14.

#### Group 6

4 Test Substance (D-TS100) patches were applied to each animal with 1 patch/week. The old patch was removed every week and new one applied. the treatment period was for 28 days (4 × 7 = 28 days). The last patch was removed on Day 29th and application site was examined for any gross pathological changes/lesions. It was followed by observation till day 35th.

**Table 1:** Experimental design, including measurement points and the type of test item used in the first step of the study.

Step 1	Group 1	Group 2	Group 3	Group 4	Group 5	Group 6
Animal species/strain/sex (Body Weight)	Minipig/male (16 ± 3 kg)					
Test item	Dapa (dapagliflozin)	Placebo ointment (D-F-P)	Dapa ointment (D-F100)	Patch-placebo (D-TS-P)	Patch Dapa (D-TS100)	Patch Dapa (D-TS100)
Number of animals	3 (MP1-3)	3 (MP4-6)	3 (MP7-9)	3 (MP1-3)	3 (MP9-12)	3 (MP13–15)
Study duration	2 Days	14 Days	14 Days	14 Days	14 Days	35 Days
Route of administration	i.v.	Topical	Topical	Topical	Topical	Topical
Feeding condition	Fed					
Dose	1 mg/kg	1 whole syringe per animal per day (Total amount = 5.9 g/Animal)	1 whole syringe per animal per day (Total amount = 5.9 g/Animal)	1 patch for 7 days	1 Patch (236 mg/patch) for 7 Days	1 patch (236 mg/patch) on day 0, day 7, day 14 and day 21
Treatment period	–	7 Days	7 Days	7 days	7 Days	28 days
Observation period	–	7 Days				
Dose volume (mL/kg)	0.5	NA				
Conc (mg/mL)/strength (mg)	2	–	47.2	–	47.2	47.2
Area (cm x cm)		6 × 6 cm	6 × 6 cm	6 × 6 cm	6 × 6 cm	6 × 6 cm
Formulation	Aqueous in NS	Ready to use				
Sample/collection—type	Blood/serial (cranial vena cava)					
Anti-coagulant	Heparin					
Sampling time points	0 h, 1 h, 2 h, 4 h, 6 h, 8 h, 12 h, 24 h and 48 h	Pre-application	0 h, 1 h, 2 h, 4 h, 6 h, 8 h, 12 h, 24 h, 48 h, 3d, 4d, 5d, 7d, 10d, 14d	Pre-application	0 h, 1 h, 2 h, 4 h, 6 h, 8 h, 12 h, 24 h, 48 h, 3d, 4d, 5d, 7d, 10d, 14d	0 h, 8d, 10d, 12d, 14d, 16d, 18d, 20d, 22d, 24d, 26d, 28d

In the second step of the experiment, a 10 × 10 cm area of skin from the dorsal region was shaved clean, washed with water and wiped with an alcohol swab to remove residual oil from the skin and to ensure good adhesion of the patch (24 h before application). The patches were removed from the refrigerator and allowed to stand at room temperature for 1–2 h before application and wiped dry. The patches were applied to clean, hairless and dry skin (a photograph of the patch application is provided in the Supplementary Appendix (Excel spreadsheet—Fig. S1). The patches were left on the skin for 2 days (group 2) or 7 days (group 1, 3 and 4). Observation was performed from day 3/8 to day 14. Detailed data on the animals and their classification into groups are presented in Table 2.

At the end of the study, all animals were euthanised by an overdose of sodium thiopental.

The study was conducted at an external certificated test facility. The test facility was certified by the Committee for the Purpose of Control and Supervision of Experiment on Animals (CPCSEA) for breeding and experimentation. The study was approved by CPCSEA and the Institutional Animals Ethics Committee (IAEC) of the testing facility. Consent was obtained from the Government of India, Ministry of Fisheries, Animal, Husbandry and Dairying, Department of Animal Husbandry and Dairying, the Committee for the Purpose of Control and Supervision of Experiments on Animals (CPCSEA) for breeding and experimentation No: 25/55/2008-AWD. This study adheres to internationally accepted standards for animal research, following the 3Rs principle. The ARRIVE guidelines were employed for reporting experiments involving live animals, promoting ethical research practices.

**Table 2:** Experimental design, including measurement points and the type of test item used in the second step of the study.

Step 2	Group 1/ST2	Group 2/ST2	Group 3/ST2	Group 4/ST2
Animal species/strain/sex (body weight)	Minipig/male (16 ± 3 kg)			
Test item	Patch 6 × 6, Dose-1 (236 mg/patch)	Patch 6 × 6, dose-1 (236 mg/patch)	Patch 6 × 6, dose-2 (118 mg/ patch)	Patch 5 × 5, dose-1 (236 mg/patch)
Number of animals	3 (MP1-3/ST2)	3 (MP4-6/ST2)	3 (MP7-9/ST2)	3 (MP9-12/ST2)
Day of treatment	Application for 7 days, observation for Day 8–14	Application for 2 days, observation for Day 3–14	Application for 7 Days, Observation for Day 8–14	Application for 7 days, observation for Day 8–14
Route of administration	Topical			
Feeding condition	Fed			
Area	6 × 6 cm 6 × 6 cm 6 × 6 cm			5 × 5 cm
Formulation	Ready to apply topical patches			
Sample/collection—type	Blood/serial (cranial vena cava)			
Anti-coagulant	Lithium Heparin			
Blood sampling time points	Main points: 0 h, 1 h, 2 h, 4 h, 6 h, 8 h, 12 h, 24 h, 2d, 3d, 4d, 5d, 7d, 10d, 14d Additionals points: 3 h, 28 h, 32 h, 36 h, 52 h, 56 h, 60 h, 78 h, 84 h, 108 h, 144 h, 192 h, 216 h, 284 h, 288 h, 312 h			
Urine collection	Main points: 24 h and 48 h of treatment initiation Additionals points: every 12 h until the end of treatment			
Urine volume	Individually recorded, for each animal at each time point			
Plasma separation	Immediately after collection			
Plasma and urine storage	Snap freezed in liquid Nitrogen, and stored at − 80 °C till subjected to bioanalysis			

### Frequency of observation

From day 1, each minipig was individually observed for abnormalities and behavioural changes (slowing, trembling, lying on the side, uncoordinated movements, abnormal gait) daily (on pre-dosing days and 2–3 h after dosing) and at least once on non-dosing/observation days. All minipigs were observed daily for mortality and morbidity and general clinical signs. All minipigs were individually observed and recorded for local skin reactions.

### Blood collection and plasma separation

In order to idenitify the pharmacokinetic parameters, the concentration of dapagliflozin in the blood plasma was determined (in steps 1 and 2) and the levels of 3-O-glucuronide of dapagliflozin (in step 2). Blood was collected from minipigs at the time points specified in Tables 1 and 2.

The following time points were used in the initial stages of the study:

- Group 1: 0 h, 1 h, 2 h, 4 h, 6 h, 8 h, 12 h, 24 h and 48 h.
- Group 3 and 5: 0 h, 1 h, 2 h, 4 h, 6 h, 8 h, 12 h, 24 h, 48 h, 3d, 4d, 5d, 7d, 10d, 14d.
- Group 6: 0 h, 8d, 10d, 12d, 14d, 16d, 18d, 20d, 22d, 24d, 26d, 28d.

In the second part of the study, the following times were used for each group: 0 h, 1 h, 2 h, 4 h, 6 h, 8 h, 12 h, 24 h, 2d, 3d, 4d, 5d, 7d, 10d, 14d as a main points.

Approximately 2 mL of whole blood was withdrawn from the cranial vena cava and collected in labelled tubes containing lithium heparin. After blood collection at each time point, blood samples were stored on ice before centrifugation. Blood samples were centrifuged within 15 min to separate plasma. Centrifugation was performed at 1540 rcf (5000 rpm), 4 °C for 10 min. Plasma was separated and transferred to pre-labelled microcentrifuge tubes and immediately frozen at − 80 ± 10 °C until bioanalysis.

### Urine collection

In the second part of the study, urine samples were collected to determine the effectiveness of the transdermal patch treatment. The levels of the active ingredient and its metabolites, as well as glucose in the urine, were measured. Urine samples were collected before treatment and 24 and 48 h after the start of treatment as a main measurement point, and additionally in parallel urine samples were collected every 12 h until the end of treatment.

Metabolic cages were used for collection. As soon as the animal urinated, the urine (approximately 2–5 ml) was transferred to a labelled plastic tube using a plastic pipette. Samples were immediately stored at approximately − 80 °C until analysis.

### Necropsy and anatomic pathology

To clearly confirm dapagliflozin transdermal transfer, dapagliflozin was evaluated in selected minipig organs. Only in the first part of the study, after day 14 for groups 2, 3, 4 and 5, and day 36th for group 6, the minipigs of the respective groups were fasted overnight and the next day, i.e. day 15 the minipigs were sacrificed by intravenous administration of thiopentone sodium and then exsanguinated (major vessel cut). The following organs were collected in filtered PBS for bioanalysis: brain, liver, heart, muscle, lung (perfused with PBS), skin and kidney for groups 2, 3, 4, 5, 6.

### Bioanalysis using LC-MS technique

Fit-for-purpose bioanalytical method was developed for analyzing the plasma and tissue samples. Chromatographic separations of the samples were performed on a Agilent 6545XT AdvanceBio LC/Q-TOF (Quadrupole Time of Flight LC/MS) chromatograph. The C18 packed column (Kinetex EVO C18 4.6 × 50 mm, 5 µl) was used, which was heated to heated to 40 °C. The volume of sample injected was 15 µl. MS and MS/MS measurements were performed in positive polarization (ESI-positive). Detailed information on the measurement method can be found in Supplementary Appendix (excel spreadsheet—Table S1).

#### Method calibration

One set of standards was run before the sample batch and was used for plotting the calibration curve. Quality control (QC) samples were prepared at a minimum of three concentrations, i.e. LQC (not more than 5 times to that of lowest standard concentration), HQC (not less than 75% of the highest standard concentration) and MQC (between the low and high concentration). Minimum of 6 QC samples (three concentrations in duplicate). One Set of QC (LQC, MQC and HQC) samples were analyzed before and after the sample batch. Calibration standards were accepted if the back-calculated concentrations did not deviate by more than 20%. Calibration data are shown in the Supplementary Appendix (Excel spreadsheet, Table S1).

### Pharmacokinetic parameters

Pharmacokinetic parameters were calculated for individual animal by Non-compartmental model with Phoenix WinNonlin software version 8.1 (Certara, USA). The software is the industry standard for pharmacokinetic (PK) analysis. The calculations have been carried out according to the book for PK/PD analysis^19^.

### Statistical analysis

All results analyzed statistically were mean values (SR) calculated from 3 independent repetitions. Parametric multivariate analysis of variance (ANOVA) with a posthoc test for multiple comparisons NIR (Fisher’s least significant difference test) was used to assess differences between means SR. In all analyses and statistical tests used, a significance level of α = 0.05 was adopted. Statistica 13.1 software (StatSoft Inc., Tulsa, OK, USA) was used for analysis.

## Results

In order to assess the pharmacokinetic parameters as well as the efficiency of skin penetration of dapagliflozin applied as ointment and patch, the concentration of dapagliflozin in the blood serum of minipigs was measured and the concentration of the active substance in selected organs of minipigs was determined. First, to establish the standard, 1 mg/kg of dapagliflozin was administered intravenously to minipigs and the pharmacokinetic parameters were determined (data are presented in Table 3). The half-life of dapagliflozin (t_**½**_) was 9.47 h and the mean residence time of the drug in the body (MRT_0 − last_) was 8 h. The next step was to compare the tested ointment and skin patch with placebo. As expected, no dapagliflozin concentrations were detected in the serum of the minipigs with either the ointment or the patch containing no active substance (placebo: group 2 and group 4). Detailed data on measurement points and concentrations determined for individual minipigs are provided in the Supplementary Appendix (Excel spreadsheet, Table S2).

**Table 3:** Plasma concentration of dapagliflozin (1 mg/kg) after *i.v.* administration in male mini-pigs (group 1) and pharmacokinetic parameters calculated for these.

Time (h)	Mean	Std dev	% CV
Plasma concentration (ng/mL)			
Predose	0.000	0.000	NA
1	548.587	101.091	18.428
2	293.007	65.403	22.321
4	213.003	93.956	44.110
6	252.657	53.280	21.088
8	125.763	28.891	22.972
12	76.780	30.674	39.950
24	25.600	17.572	68.641
48	7.100	7.664	107.948
Pharmacokinetic parameters			
Dose (mg/kg)	1	0	0
C_0_ (ng/mL)	1130.714	543.869	48.100
t_½_ (h)	9.474	2.491	26.290
Vss (L/kg)	2.303	0.456	19.796
Vd (L/kg)	3.456	0.118	3.424
Cl (mL/min/kg)	4.374	0.916	20.937
AUC_last_ (ng×h/mL)	3825.606	791.548	20.691
AUC_inf_ (ng×h/mL)	3940.873	937.350	23.785
AUC_Extra_ (%)	2.492	2.734	109.744
AUC_inf_D_ (h×kg×ng/mL/mg)	3940.873	937.350	23.785
MRT_0 − last_ (h)	8.008	2.694	33.645
Rsq	0.985	0.006	0.560

When dapagliflozin ointment and transdermal patch were used in both the ointment (group 3) and patch (group 5) groups, it was not possible to confirm the presence of dapagliflozin in serum at levels that could be considered significant. The mean C_max_ for the ointment was 1.80 ng/mL with a tmax of 112 h, while the mean C_max_ for the patch was 5.73 ng/mL with a t_max_ of 72 h. Therefore, it can be concluded that a higher concentration of dapagliflozin is achieved in the blood serum of minipigs following patch application. The results were statistically significant (*p* < 0.05). When assessing the organ accumulation of dapagliflozin in both groups (groups 3 and 5), no statistically significant differences were observed in most organs (*p* > 0.05). Mean data are presented in Table 4, and detailed data are presented in the Supplementary Appendix (Excel spreadsheet, Table S2).

**Table 4:** Comparison of the mean results of plasma concentration of dapagliflozin at each measurement point for ointments (group 3) and transdermal patches (group 5), as well as mean pharmacokinetic parameters for both groups and mean dapagliflozin tissue accumulation.

Time (h)	Dapa ointment (D-F100) (Group 3)			Patch Dapa (D-TS100) (Group 5)		
	Mean	Std dev	% CV	Mean	Std dev	% CV
Plasma concentration (ng/mL)						
Predose	0	0	NA	0	0	NA
1	NC	NA	NA	NC	NA	NA
2	NC	NA	NA	NC	NA	NA
4	NC	NA	NA	NC	NA	NA
6	NC	NA	NA	NC	NA	NA
8	NC	NA	NA	NC	NA	NA
12	NC	NA	NA	NC	NA	NA
24	NC	NA	NA	NC	NA	NA
48	1.560	0.099	6.346	2.400	2.107	87.799
72	NC	NA	NA	NC	NA	NA
96	1.790	0.594	33.183	5.380	3.111	57.830
120	1.523	0.410	26.925	NC	NA	NA
168	1.627	0.424	26.041	NC	NA	NA
240	NC	NA	NA	NC	NA	NA
336	3.165	2.949	93.164	NC	NA	NA
Pharmacokinetic parameters						
Dose (mg/kg)	5.432	0.348	6.415	5.564	0.883	15.862
C_max_ (ng/mL)	1.807	0.421	23.302	5.735	2.609	45.496
tmax (h)	112.000	13.856	12.372	72.000	33.941	47.140
t_½_ (h)	NC	NA	NA	NC	NA	NA
AUC_last_ (ng×h/mL)	336.624	163.910	48.692	234.194	39.986	17.074
AUC_inf_ (ng×h/mL)	NC	NA	NA	NC	NA	NA
AUC_Extra_ (%)	NC	NA	NA	NC	NA	NA
AUC_inf _D_ (h×kg×ng/mL/mg)	NC	NA	NA	NC	NA	NA
MRT_0 − last_ (h)	134.964	24.811	18.383	63.360	0.588	0.929
Rsq	NC	NA	NA	NC	NA	NA
F (%)	1.620	0.789	48.692	1.100	0.188	17.074

Tissue matrix	Time (h)	Mean	SD	Time (h)	Mean	SD
Tissue concentration (ng/g)						
Brain	336	NC	NA	336	NC	NA
Liver		14.550	2.192		NC	NA
Heart		15.383	7.080		13.400	6.223
Skeletal muscles		101.750	76.179		116.525	38.785
Lungs		34.383	16.974		21.400	18.416
Kidneys		131.183	69.975		25.600	16.982
Skin (treated area)		15448.000	8232.690		15773.267	3727.420
Adipose tissue		309.033	130.054		94.367	23.936

Furthermore, the repeated application of the patch was found to be crucial in terms of the accumulation of dapagliflozin in the organs (group 6). The results demonstrated that repeated application of the patch significantly increased the drug reservoir in the skin and other organs (*p* < 0.05) in comparison to group 5. The data are presented in Table 5 and in the Supplementary Appendix (Excel spreadsheet, Table S2).

**Table 5:** Mean tissue accumulation of dapagliflozin after repeated application of the dapagliflozin patch (Group 6).

Tissue concentration (ng/g)			
Tissue matrix	Time (h)	Concentration (ng/g)	
		Mean	SD
Brain	672	213.000	238.162
Liver		229.667	180.506
Heart		675.000	858.112
Skeletal muscles		310.000	224.012
Lungs		3444.167	2187.830
Kidneys		303.333	161.909
Skin (treated area)		43,092.500	38,151.822
Adipose tissue		545.000	474.151

Following the confirmation of tissue accumulation of dapagliflozin and the potential for absorption through the skin, the second phase of the study was initiated to assess the absorption and kinetics of dapagliflozin using transdermal patches of different strengths and different treatment durations. The assessment was extended to measure the concentration of dapagliflozin and its metabolite dapagliflozin 3-O-glucuronide in both urine and plasma. In addition, glucose concentrations were measured in the urine of minipigs to further confirm the efficacy of transdermal delivery. Based on the main measured parameters, pharmacokinetic parameters were determined for each of the groups tested. The results are summarised in Tables 6 and 7.

**Table 6:** Comparison of the mean results of the concentration of dapagliflozin and its metabolite in the plasma of male minipigs for group 1/ST2 and group 2/ST2 and the mean pharmacokinetic parameters determined for both groups.

Step 2	Group 1/ST2						Group 2/ST2					
	Dapagliflozin			Dapa 3-O-gluc.			Dapagliflozin			Dapa 3-O-gluc.		
Time (h)	Mean	Std dev	% CV	Mean	Std dev	% CV	Mean	Std dev	% CV	Mean	Std dev	% CV
Plasma concentration (ng/mL)												
Predose	0	0	NA	0	0	NA	0	0	NA	0	0	NA
1	NC	NA	NA	NC	NA	NA	NC	NA	NA	0.57	0.15	26.28
6	NC	NA	NA	0.57	0.16	27.29	1.87	1.51	80.76	1.84	0.67	36.61
12	31.79	52.19	164.17	12.17	19.51	160.34	4.03	2.74	68.08	2.21	2.16	97.83
24	52.65	80.2	152.33	19.74	27.3	138.32	4.55	3.33	73.08	5.49	5.23	95.13
48	10.23	9.36	91.57	3.68	2.33	63.33	4.52	1.7	37.57	4.15	2.39	57.62
72	3.55	0.81	22.8	1.46	0.37	25.45	2.07	1.08	52.21	2.44	1.93	79.24
96	3.46	1.9	54.96	1.26	0.37	29.53	1	0.45	44.77	1.36	0.7	51.8
168	2.99	2.49	83.24	1.28	1.03	80.81	NC	NA	NA	0.6	0.08	14.14
240	NC	NA	NA	NC	NA	NA	NC	NA	NA	0.62	0.01	1.15
336	NC	NA	NA	NC	NA	NA	NC	NA	NA	NC	NA	NA
Pharmacokinetic parameters												
C_max_ (ng/mL)	52.82	80.05	151.54	19.83	27.21	137.18	5.26	2.63	50.03	5.91	4.81	81.36
t_max_ (h)	32	13.86	43.3	32	13.86	43.3	32	13.86	43.3	32	13.86	43.3
t_½_ (h)	70.86	14.46	20.41	108.28	41.8	38.6	NC	NA	NA	201.17	188.22	93.57
AUC_last_ (ng×h/mL)	1684.31	2104.04	124.92	709.02	790.09	111.43	272.49	145.37	53.35	408.84	279.96	68.48
AUC_inf_ (ng×h/mL)	2704.8	2767.68	102.32	821.81	842.22	102.48	NC	NA	NA	562.75	407.72	72.45
AUC_Extra_ (%)	13.15	1.75	13.28	19.67	8.74	44.42	NC	NA	NA	25.42	8.79	34.59
MRT_0 − last_ (h)	52.52	20.68	39.38	68.07	1.24	1.81	43.09	8.87	20.58	79.88	3.94	4.94
Rsq	0.4904	0.36	72.55	0.7915	0.34	42.78	NC	NA	NA	0.763	0.32	42.02

**Table 7:** Comparison of the mean results of the concentration of dapagliflozin and its metabolite in the plasma of male minipigs for group 3/ST2 and group 4/ST2 and the mean pharmacokinetic parameters determined for both groups.

Step 2	Group 3/ST2						Group 4/ST2					
	Dapa			Dapa 3-O-gluc.			Dapa			Dapa 3-O-gluc.		
Time (h)	Mean	Std Dev	% CV	Mean	Std dev	% CV	Mean	Std dev	% CV	Mean	Std dev	% CV
Plasma concentration (ng/mL)												
Predose	0	0	NA	0	0	NA	0	0	NA	0	0	NA
1	NC	NA	NA	NC	NA	NA	NC	NA	NA	NC	NA	NA
6	1.1	0.47	42.29	0.79	0.37	47.74	1.09	0.81	74.95	0.51	0.05	9.8
12	2.07	0.98	47.46	1.37	0.84	61.36	1.93	0.17	8.81	1.29	0.21	15.96
24	17.16	23.34	136.04	13.53	18.75	138.6	6.41	3.81	59.52	2.76	0.86	31.18
48	7.92	7	88.35	6.02	5.26	87.25	7.94	2.68	33.81	5.43	1.48	27.3
72	3.84	1.9	49.5	3.07	0.99	32.31	8.45	1.55	18.34	5.78	1.08	18.75
96	2.15	0.87	40.45	1.48	0.76	51.24	129.99	214.8	165.2	54.18	86.88	160.4
168	1.29	0.17	13.16	1.33	0.4	29.77	15.67	21.59	137.8	9.41	11.79	125.3
240	0.81	0.32	39.53	NC	NA	NA	5.86	6.55	112	2.08	2.57	123.6
336	NC	NA	NA	NC	NA	NA	NC	NA	NA	NC	NA	NA
Pharmacokinetic parameters												
C_max_ (ng/mL)	17.5	23.03	131.64	13.74	18.56	135.04	131.91	213.14	161.6	55.67	85.58	153.7
t_max_ (h)	32	13.86	43.3	32	13.86	43.3	64	36.66	57.28	80	13.86	17.32
t_½_ (h)	65.98	44.81	67.91	86.17	66.96	77.71	49.79	25.46	51.13	52.84	6.2	11.73
AUC_last_ (ng×h/mL)	746.4	480.32	64.35	542.64	392.35	72.3	6620.7	9712.96	146.7	3144.2	4210.84	133.9
AUC_inf_ (ng×h/mL)	831.3	447.16	53.79	712.11	300.3	42.17	6728.6	9633.71	143.2	759.01	110.72	14.59
AUC_Extra_ (%)	13.01	9.43	72.47	28.65	23.67	82.62	8.8	7.55	85.76	6.12	1.81	29.53
MRT_0 − last_ (h)	76.21	32.42	42.54	65.02	23.48	36.11	101.18	23.8	23.53	108.04	11.89	11
Rsq	0.9608	0.03	3.08	0.824	0.29	35.07	0.937	0.09	9.64	0.9015	0.06	6.19

The values obtained for additional measurement points (according to Table 2) and the pharmacokinetic parameters determined on this basis are presented in the Supplementary Appendix (excel spreadsheet Table S4). It can be seen that group 4/ST2 achieves significantly higher parameters both in terms of AUC and C_max_ (*p* < 0.05). Therefore, this formulation ensures an effective transfer of dapagliflozin through the skin, but also a relatively long residence time of the drug in the body of minipigs.

**Fig. 1:**
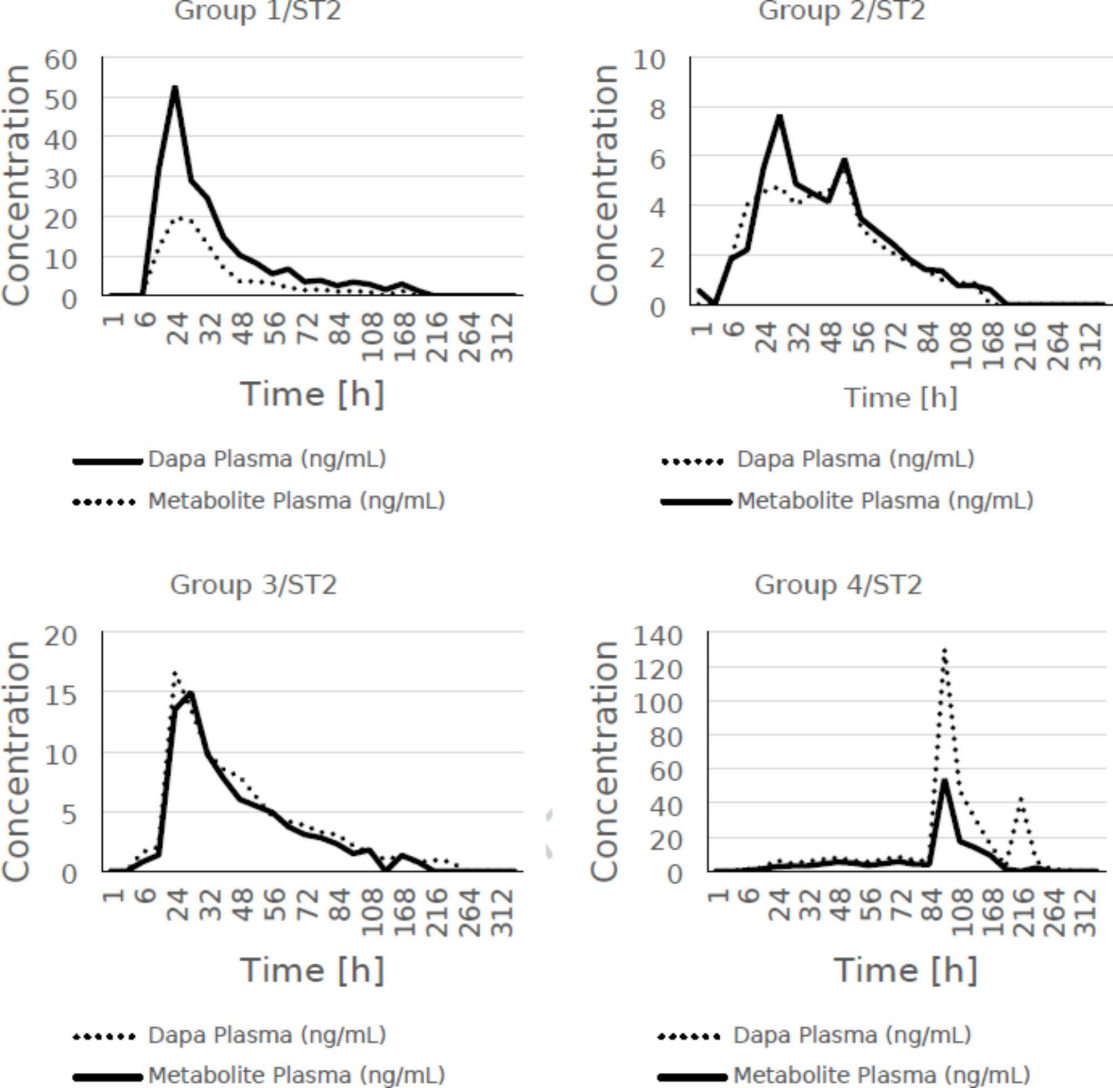
Comparison of the mean concentration of dapagliflozin and its metabolite in the plasma of minipigs for individual groups, taking into account all measurement points (main and additional).

**Fig. 2:**
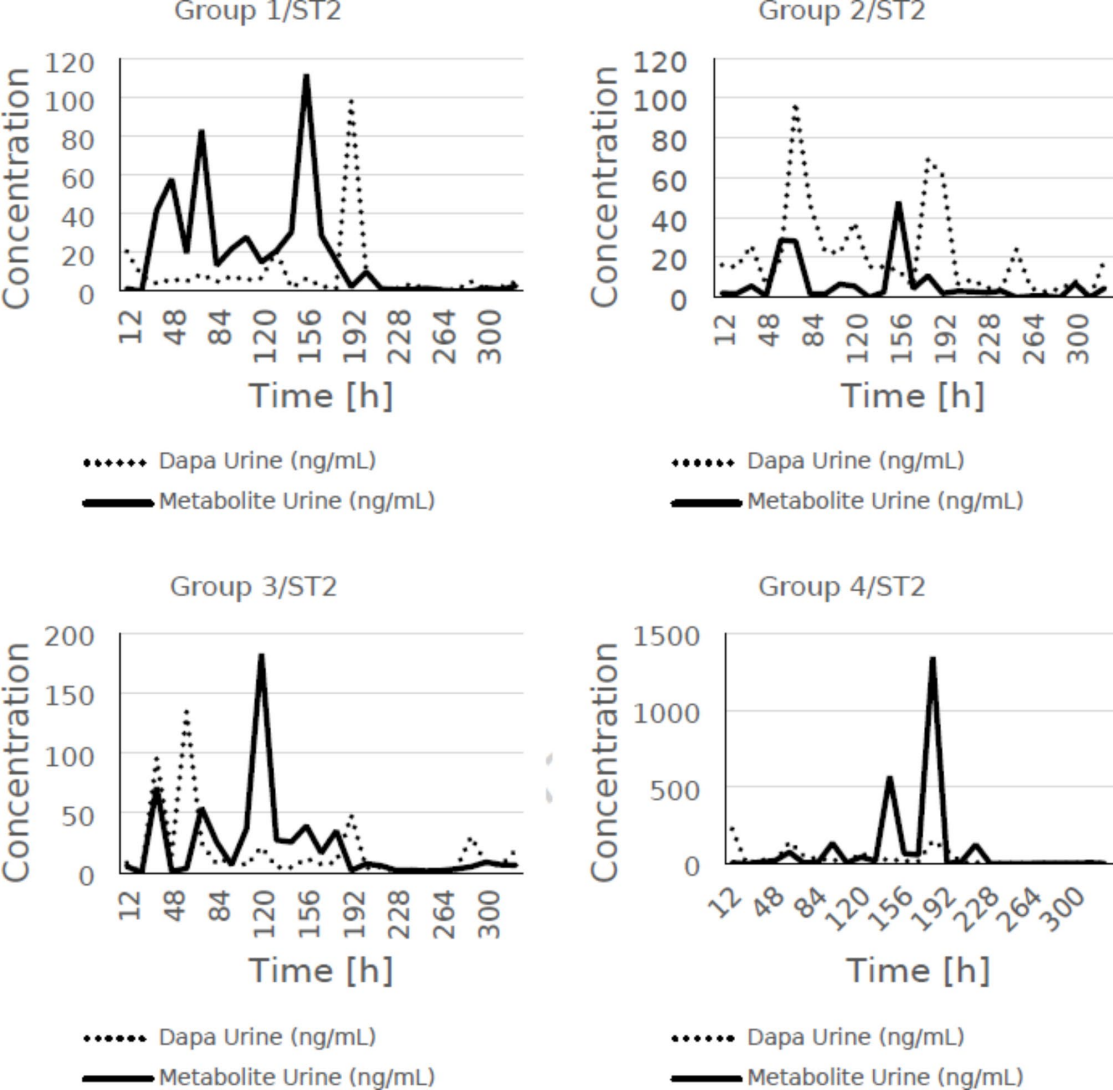
Comparison of the mean concentration of dapagliflozin and its metabolite in the urine of minipigs for individual groups, taking into account all measurement points (main and additional).

**Fig. 3:**
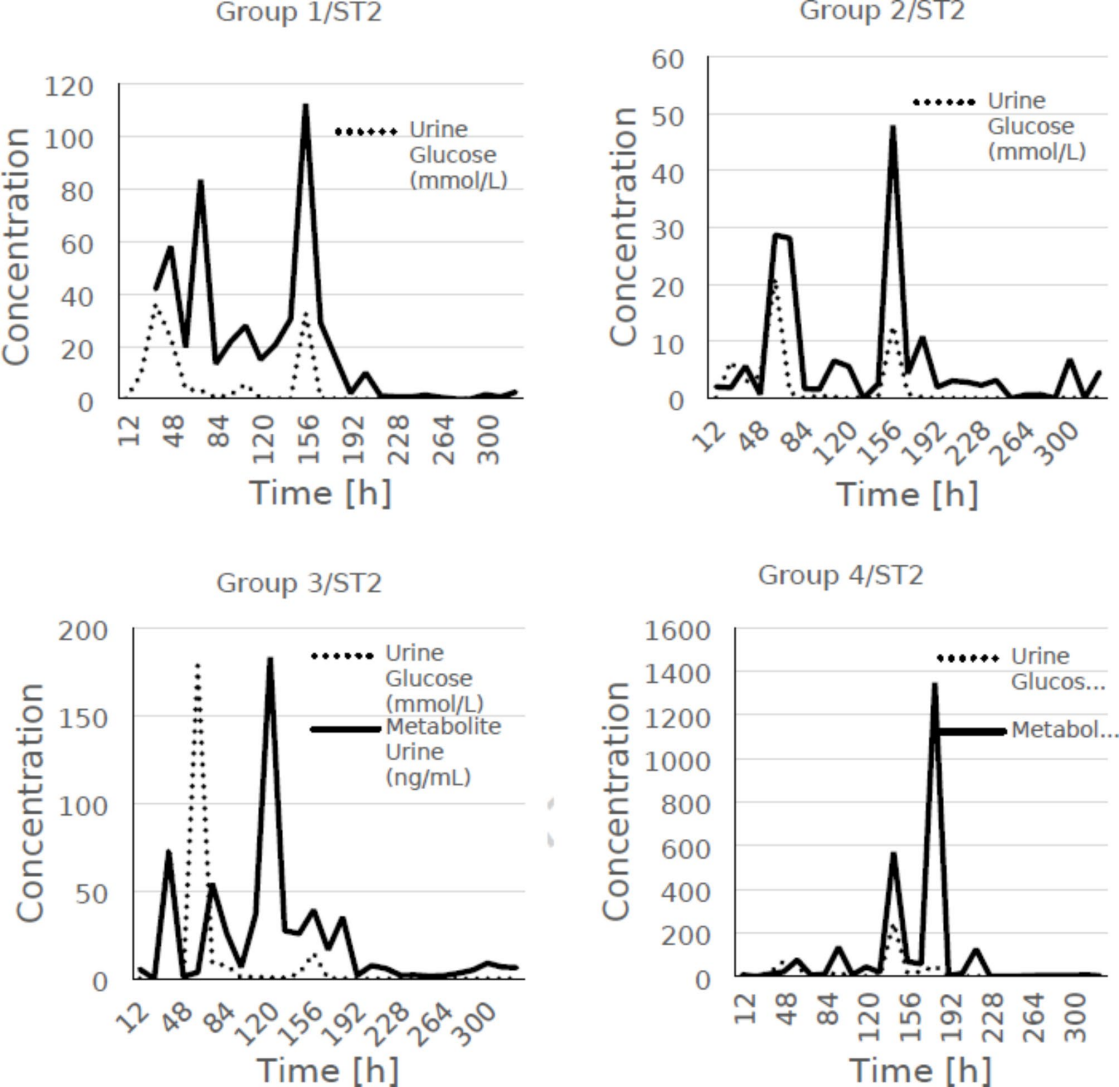
Comparison of mean urinary excretion of dapagliflozin metabolites and glucose in each group of minipigs, taking into account all measurement points (main and additional).

A comparative analysis of dapagliflozin 3-O-glucuronate levels in urine and urinary glucose excretion over time was performed for the main time points. A trend was observed between dapagliflozin 3-O-β-D-glucuronide levels in urine and urinary glucose excretion. The data for the main points are summarised in the Table 8. However, analogous comparisons are shown in Figs. 1, 2 and 3 for all measured points (including main and additional points). It is noteworthy that the amount of glucose excreted in the urine increased significantly (*p* < 0.05) in all groups 48 h after application, except in group 2.

**Table 8:** Comparison of the mean concentrations of dapagliflozin, its metabolite and glucose in the urine of minipigs in each of the groups tested.

	Time [h]	Group 1/ST2			Group 2/ST2		
		Mean	Std dev	% CV	Mean	Std dev	% CV
Dapagliflozin (ng/mL)	24	7.97	9.44	118.39	15.13	7.72	51.02
	48	6.18	3.94	63.75	6.03	2.64	43.82
Dapagliflozin 3-0-glucuronide (ng/mL)	24	217.55	274.86	126.34	NC	NA	NA
	48	58.10	68.45	117.81	0.68	NA	NA
Glucose (mMol/L)	24	9.07	10.30	113.56	6.45	NA	NA
	48	23.32	26.82	114.99	3.91	3.79	96.85

	Time [h]	Group 3/ST2			Group 4/ST2		
		Mean	Std dev	% CV	Mean	Std dev	% CV
Dapagliflozin (ng/mL)	24	NC	NA	NA	NC	NA	NA
	48	14.84	18.92	127.46	12.56	11.43	90.98
Dapagliflozin 3-0-glucuronide (ng/mL)	24	NC	NA	NA	NC	NA	NA
	48	1.36	0.81	59.56	21.12	25.76	121.95
Glucose (mMol/L)	24	0.10	NA	NA	NC	NA	NA
	48	0.19	4.67	2415.52	68.77	45.43	66.06

The results of the above determinations for individual minipigs and the results of determinations including additional measurement points are presented in the Supplementary Appendix (Excel sheet, Tables S5-S7).

Taking into account all measurement points, the concentrations of dapagliflozin and its metabolite in plasma were tabulated and compared. Plasma dapagliflozin 3-O-glucuronate concentrations were detectable in parallel with dapagliflozin concentrations after the application of the dapagliflozin patch. The kinetics of dapagliflozin in plasma are broadly consistent with those of dapagliflozin 3-O-glucuronate, as shown in Fig. 1 and the data in Tables 6 and 7.

A comparison was made to determine the urinary excretion trends of dapagliflozin and dapagliflozin 3-O-glucuronide and the results are presented below. Overall, the concentration of the more polar metabolite dapagliflozin-3-O-glucuronide was found to be higher than that of dapagliflozin in the urine of minipigs in groups 1, 3 and 4. However, in group 2, dapagliflozin concentrations were higher at many time points, but the duration of dapagliflozin administration in this group was shorter (2 days).

Taking into account that both the site of action of dapagliflozin and the site of the main metabolising enzyme UGT1A9 are located in the proximal tubules of the renal cortex, a comparative analysis of the levels of dapagliflozin 3-O-glucuronate in urine and urinary glucose excretion over time was performed and trend graphs are presented below. This is shown in Fig. 3; Table 8.

The data confirm the efficacy of transdermal delivery of dapagliflozin and the efficacy of the drug when used as a transdermal patch.

## Discussion

Given the extended timeframe of diabetes treatment, a transdermal delivery system is expected to be a more suitable option, particularly in terms of patient compliance. The most common form of non-compliance observed in older people on long-term chronic therapy is underdosing. Transdermal patches are more patient- and caregiver-friendly, allowing diabetics to remain on therapy longer and achieve greater and more sustained glucose homeostasis.

Administering dapagliflozin in a non-oral form, i.e. as an ointment or patch, as a new drug formulation is a major challenge, particularly if it is to be as effective and comparable as oral administration. That’s why the study was designed to determine the safety and pharmacokinetics of dapagliflozin in the blood plasma of male minipigs after application of the ointment and patch to the skin, to determine the concentration in the skin layers and soft tissues (Step 1), and to assess the efficacy of using dapagliflozin in patches of different strengths and sizes (Step 2). A total of 27 minipigs were tested (15 in Step 1 and 12 in Step 2).

The most important requirement in the application of transdermal drugs is that the skin tolerates the treatment. No local skin reactions were observed in any of the groups of animals tested. This indicates that the formulation is completely safe and well tolerated. The possibility of potential damage to the transdermal patch matrix (STEP1 - group3) was also investigated as the ointment tested was a filler of the transdermal patch and no local skin reactions were observed. The calculated maximum concentration for the ointment (C_max_ = 1.807 ng/ml at t_max_ 112 h) is significantly lower than for the transdermal patch (C_max_ = 5.735 ng/ml at t_max_ 72 h) (Table 4), confirming that the preparation is safe and does not cause overdosage in case of patch matrix damage.

In the first part of the study in *i.v.* group 1, the mean t_½_ after *i.v.* administration of dapagliflozin was approximately 9.47 h (Table 3), close to the reported value of 12.2 h. Although substantial plasma levels were not detectable at defined time intervals in either the patch or ointment groups, quantifiable tissue levels were evident, indicating that transdermal absorption was indeed occurring in both the ointment and patch groups. Significant levels remain in the skin even after 7 days of treatment discontinuation (15448.0–15773.0 ng/g), indicating drug retention in the skin (Table 4). Group 6 (D-TS100), 1 patch/week x 4 weeks showed the highest dapagliflozin levels (ng/g) in heart, skeletal muscles, lungs, kidneys, skin and adipose tissue (Table 5) compared to the other transdermal intervention groups (patch or ointment).

The Part II study was designed to evaluate the extent of absorption and the kinetics of dapagliflozin when transdermal patches of different strengths were applied for different durations.

After topical patch application of dapagliflozin in group-1, a C_max_ of 55.82 ng/mL was observed at 24–32 h with a mean AUC_0 − last_ of 1684.31 ng × hr/mL (Table 6). The first concentration time point was observed at 12 h, suggesting that dapagliflozin released from the patch formulation was able to penetrate the skin layers quite efficiently and reach the systemic circulation. Although the patches were applied for 7 days (168 h), final concentrations (0.74 ng/mL) were detectable until 192 h (8 days), suggesting a depot/reservoir in the skin (Table S4).

After topical patch application of dapagliflozin in group 2, a C_max_ of 5.26 ng/mL was observed at 28–52 h with a mean AUC_0 − last_ of 272.49 ng × h/mL (Table 6). The first peak was observed at 3 h in the third animal, 6 h in the second animal and 24 h in the third animal, again suggesting that dapagliflozin was rapidly absorbed from the patches and was able to rapidly enter the systemic circulation (Table S4). The patches were only applied for 2 days (48 h) but the final concentrations (0.87 ng/ml) could be quantified up to 6 days (144 h), indicating a long mean residence time of dapagliflozin in the skin (Table S4).

Following topical patch application of dapagliflozin in group 3, a C_max_ of 17.50 ng/mL was observed at 24–56 h with a mean AUC_0 − last_ of 706.4 ng × h/mL (Table 7). Peak concentrations were observed at 3 h in the second animal, 6 h in the first animal and 24 h in the third animal, indicating that dapagliflozin re-release from the formulation and permeation and absorption through the skin is quite rapid and efficient. Dapagliflozin was detectable in the systemic circulation within 3 to 6 h after patch application, which is expected to facilitate rapid onset of action despite the topical/dermal nature of the intervention (Table S4). The patches were applied for only 7 days (168 h), but terminal concentrations (0.81 ng/mL) of dapagliflozin were observed up to 10 days (240 h), suggesting depot/reservoir formation followed by slow and prolonged release from the skin layers (Table 7).

After topical patch application of dapagliflozin in group 4, the highest C_max_ (131.91 ng/mL) compared to the other groups with a mean AUC_0 − last_ of 6620.7 ng × h/mL (Table 7). The first concentration was quantifiable at 3 h in the 3rd animal, 6 h in the 2nd and 12 h in the 3rd animal, reinforcing the notion that dapagliflozin is effectively released from the formulation and efficiently penetrates through the skin layers to become available in the systemic circulation within 3 to 12 h after patch application (Table S4, Table 7). Although the treatment lasted only 7 days (168 h) and the patches were removed thereafter, terminal concentrations (0.64 ng/mL) were observed up to 12 days (288 h) and for 2nd animal up to 14 days (336 h), indicating a long mean skin residence time and prolonged permeation of dapagliflozin across skin layers (Table S4).

The concentrations of dapagliflozin 3-O-glucuronide in plasma were detectable in parallel with that of dapagliflozin after dapagliflozin patch application, suggesting concomitant metabolism of dapagliflozin. The C_max_ of dapagliflozin 3-O-glucuronide was 19.83, 5.91, 13.74 and 55.67 ng/mL and the AUC_0 − last_ was 709.02, 408.84, 542.64 and 3144.2 ng × h/mL for groups 1, 2, 3 and 4, respectively (Tables 6 and 7). Interestingly, the plasma kinetics trend of dapagliflozin was found to be largely harmonious with that of dapagliflozin 3-O-glucuronide, with dapagliflozin plasma concentrations at times equalling or exceeding those of dapagliflozin 3-O-glucuronide (Fig. 1).

This is in contrast to the oral kinetics of dapagliflozin, where the proportion of circulating inactive metabolite dapagliflozin 3-O-glucuronide is much higher than dapagliflozin due to extensive metabolism by liver, colon and small intestine resident UGT1A9s^8,10^. This confirms that a major metabolic checkpoint has indeed been bypassed by the transdermal approach and that much less drug would be required in the patch to achieve the therapeutic efficacy of a 5 mg or 10 mg tablet.

As both the site of action of dapagliflozin and the site of the major metabolising enzyme UGT1A9 are the same, i.e. the proximal tubular cells of the renal cortex, a comparative analysis of dapagliflozin 3-O-glucuronide levels in urine and urinary glucose excretion over time was performed.

In general, there was a trend between urinary dapagliflozin 3-O-glucuronide levels and urinary glucose excretion. Urinary dapagliflozin 3-O-glucuronide kinetics were largely consistent with urinary glucose excretion, further indicating the efficacy of therapy (Fig. 3).

Finally, a comparison was made to determine the trend of dapagliflozin and dapagliflozin 3-O-glucuronide excretion in urine. The more polar metabolite dapaglflozin-3-O-glucuronide was generally found to be more abundant than dapagliflozin in the urine of group 1, 3 and 4 minipigs. Predominantly, the elimination trend of dapagliflozin was analogous to the elimination pattern of dapagliflozin-3-O-glucuronide, with concentration peaks of later more profound than earlier in groups 1, 3 and 4. In group 2, dapagliflozin concentrations were higher at several time points, which may be due to the high patch strength (higher dapagliflozin drug content) combined with the bypassing of major metabolic checkpoints (liver and small intestine) via the transdermal route (Fig. 2).

The study therefore provides information on the potential for dapagliflozin to be transferred through the skin and provides opportunities for further work on a carrier system for dapagliflozin to provide an alternative to the oral form.

## Conclusion

It has been confirmed and demonstrated that the transfer of dapagliflozin through the skin is possible and effective. The transdermal patch was found to be an effective drug delivery system for dapagliflozin, which, when administered in such a carrier, penetrates the skin barrier and enters the bloodstream. The maximum drug concentration (122.99 ng/ml) in plasma was observed on the fourth day of application. It has been confirmed that dapagliflozin reaches soft tissues (muscles) and parenchymal organs, including the kidneys, which have the highest number of SGLT2 receptors, through the blood. Following transdermal administration, dapagliflozin is excreted in the urine, where its presence has been detected for up to 7 days after application. The results are promising enough to allow the next stage of clinical trials to be planned and represent an important step in the development of modern anti-diabetic therapy.

## Electronic supplementary material

Below is the link to the electronic supplementary material.

Supplementary Material 1

## Data Availability

All data generated or analysed during this study are included in this published article and its supplementary information files.
